# Glucocorticoids suppress Wnt16 expression in osteoblasts *in vitro* and *in vivo*

**DOI:** 10.1038/s41598-018-26300-z

**Published:** 2018-06-07

**Authors:** Susanne Hildebrandt, Ulrike Baschant, Sylvia Thiele, Jan Tuckermann, Lorenz C. Hofbauer, Martina Rauner

**Affiliations:** 1 Division of Endocrinology, Diabetes, and Bone Diseases, Department of Medicine III, Technische Universität Dresden, Dresden, Germany; 2 Center for Healthy Aging, Technische Universität Dresden, Dresden, Germany; 3 Institute of Comparative Molecular Endocrinology (CME), University of Ulm, Ulm, Germany; 4 Present Address: Institute of Chemistry and Biochemistry, Freie Universität Berlin, Berlin, Germany

**Keywords:** Mechanisms of disease, Bone

## Abstract

Glucocorticoid-induced osteoporosis is a frequent complication of systemic glucocorticoid (GC) therapy and mainly characterized by suppressed osteoblast activity. Wnt16 derived from osteogenic cells is a key determinant of bone mass. Here, we assessed whether GC suppress bone formation via inhibiting Wnt16 expression. GC treatment with dexamethasone (DEX) decreased *Wnt16* mRNA levels in murine bone marrow stromal cells (mBMSCs) time- and dose-dependently. Similarly, Wnt16 expression was also suppressed after DEX treatment in calvarial organ cultures. Consistently, mice receiving GC-containing slow-release prednisolone pellets showed lower skeletal *Wnt16* mRNA levels and bone mineral density than placebo-treated mice. The suppression of *Wnt16* by GCs was GC-receptor-dependent as co-treatment of mBMSCs with DEX and the GR antagonist RU-486 abrogated the GC-mediated suppression of *Wnt16*. Likewise, DEX failed to suppress *Wnt16* expression in GR knockout-mBMSCs. In addition, *Wnt16* mRNA levels were unaltered in bone tissue of GC-treated GR dimerization-defective *GR*^*dim*^ mice, suggesting that GCs suppress *Wnt16* via direct DNA-binding mechanisms. Consistently, DEX treatment reduced *Wnt16* promoter activity in MC3T3-E1 cells. Finally, recombinant Wnt16 restored DEX-induced suppression of bone formation in mouse calvaria. Thus, this study identifies Wnt16 as a novel target of GC action in GC-induced suppression of bone formation.

## Introduction

Glucocorticoid-induced osteoporosis (GIO) is the most common form of secondary osteoporosis and affects up to 50% of patients receiving long-term glucocorticoid (GC) therapy^1–3^. GIO is characterized by a systemic loss of bone mass, microarchitecture and strength, leading to an increased risk of fractures^4–8^. Due to their potent immunosuppressive characteristics, GCs are widely used for the systemic treatment of inflammatory disorders such as rheumatoid arthritis, vasculitis, or inflammatory bowel disease. Thus, there is a great need to further define the underlying mechanisms of GIO to specifically target pathways to prevent GIO and the subsequent increase in fracture risk.

GIO is characterized by a rapid stimulation of bone resorption, followed by a sustained and profound suppression of bone formation^9–11^. While physiological concentrations of GCs stimulate osteoblast differentiation^12^, pharmacological doses inhibit osteoblastogenesis^13,14^. GCs inhibit bone formation by suppressing pro-osteoblastic genes such as the key pro-osteogenic transcription factor Runx2^15,16^ and osteocalcin^17,18^, osteoblast-derived pro-osteogenic growth factors and cytokines such as insulin growth factor 1 (IGF-1)^19^ and IL-11^13^ as well as by suppressing collagen I synthesis^16,20–22^.

In recent years, the Wnt signaling pathway has emerged as a major regulator of bone homeostasis^23^. Activation of the Wnt pathway leads to high bone mass in mice and humans, whereas inactivation of Wnt signaling leads to low bone mass^23–25^. However, to date the function of specific Wnt ligands in bone homeostasis has not yet been completely clarified. Wnt16 is a relatively young member of the Wnt family and is expressed in skeletal tissues^26,27^. Genome-wide association studies have linked missense mutations in the *WNT16* gene to lower cortical bone thickness, reduced bone mineral density and increased fracture risk in humans^28–33^. The regulatory role of Wnt16 on cortical bone and bone strength was further validated using global and osteoblast-lineage *Wnt16* knock-out mice^32,34,35^, showing that osteoblast-derived Wnt16 inhibits osteoclastogenesis^34^. Overexpression of *Wnt16* in osteoblasts or osteocytes led to higher cortical as well as trabecular bone mass in mice and caused an increase in bone formation compared to controls^36,37^ suggesting that Wnt16 also promotes osteoblast differentiation and function, similar to other Wnt ligands^38^.

Several studies indicate that GCs suppress canonical Wnt signaling by reducing various signalling components such as cytosolic and nuclear abundance of β-catenin^16,39,40^ and gene expression of low-density lipoprotein 5^16,41^. Concurrently, GCs increase the expression of Wnt inhibitors such as Dickkopf-1^16,42,43^ and Wnt inhibitor factor 1^44,45^ suggesting that the inhibition of Wnt signaling may be a key mechanism of GC-induced suppression of bone formation. Despite the crucial role of Wnt16 in the regulation of bone homeostasis, the modulation of Wnt16 by GCs has not been studied so far. Thus, in this study, we tested the hypothesis that GCs inhibit bone formation via suppressing Wnt16 expression.

## Methods

### Declaration of approval for animal experiments

All experiments involving animals were approved by the Landesdirektion Sachsen, Germany and were performed in accordance with the relevant guidelines and regulations (approval number: TVV 86/2017).

### Animals and induction of glucocorticoid-induced bone loss

Full GR knock-out mice (*GR*^*−/−*^) and mice with impaired GR dimerization (*GR*^*dim*^) were described previously^13^. Two different models of GC-induced bone loss were performed. In the first experiment, 24-week-old female FVB/N mice were used and received a 3.5 mg prednisolone (n = 6) or placebo (n = 6) pellet (60 day release; Innovative Research of America, Inc.) for 4 weeks. In the second experiment, 10-week-old female FVB/N mice received slow-release pellets with prednisolone (PRED, n = 3) or placebo (n = 3) for 14 days (15 mg; 60 day release; Innovative Research of America, Inc.). Pellets were implanted subcutaneously and released a calculated prednisolone dose of 12.5 mg/kg/day. This resulted in a calculated dose of 2 mg/kg/day. Bone loss and suppression of bone formation was confirmed in both models and has been reported previously^13,18^. Animal protocols were approved by the Landesdirektion Sachsen, Germany.

### Bone analysis

Trabecular bone mineral density was assessed in the distal femur by peripheral quantitative computed tomography (pQCT; Stratec, Pforzheim, Germany). The measurements were made with a voxel size of 70 µm at the distal femur (two slices at the trabecular region located 1 mm from the growth plate, one at the mid-diaphysis located 6 mm from the growth plate). For the measurement of trabecular bone mineral density, regions of interest were set. For contouring mode 1 and peel mode 20 were used. Quality control measurements were performed using the standard and cone phantom, which was supplied by the manufacturer.

### *In vivo* calvarial model

Male 12-week-old C57BL/6j wildtype mice were injected subcutaneously with 0.5 mg/kg dexamethasone (DEX, Sigma-Aldrich) or 0.5 mg/kg DEX and 500 ng recombinant human WNT16 (Peprotech) three times per week, respectively, in a total volume of 25 µl. One group received 25 µl PBS injections as vehicle control. Five and 2 days before sacrifice, the mice received two intraperitoneal injections of calcein (20 mg/kg, Sigma-Aldrich). The mice were sacrificed after three weeks. Calvariae were fixed in 4% PBS-buffered paraformaldehyde and dehydrated in ascending ethanol series. Subsequently, calvariae were embedded in methacrylate and cut into 7 µm sections to assess fluorescence labels using fluorescence microscopy. Bone formation rate per bone surface (BFR/BS) was determined using the Osteomeasure software (OsteoMetrics, Decatur, GA, USA).

### Osteoblast cultures

Bone marrow was flushed from hind legs of C57BL/6 or GR mutant mice and seeded at a density of 1,000,000 cells per cm² in DMEM containing 10% fetal calf serum (FCS) and 1% penicillin/streptomycin (Pen/Strep) (all from Invitrogen, Karlsruhe, Germany). At 70% confluence, cells were switched to differentiation media with 5 mM β-glycerol phosphate and 100 µM ascorbate phosphate (all from Sigma-Aldrich) for 7 days. Thereafter, cells were switched to starving media (DMEM containing 1% FCS and 1% Pen/Strep) overnight and treated with DEX (Sigma-Aldrich) at concentrations as indicated in the figure legends for a maximum of 48 hours. In some experiments, cells were co-treated with 1 µM RU-486 (Sigma-Aldrich), an established GR antagonist.

### Calvaria organ culture

Calvarial bones were isolated from 3–5 day old C57BL/6 pups and surrounding tissue was removed. Both halves were placed in a culture dish with DMEM containing 10% FCS and 1% Pen/Strep. Calvariae were stimulated with 1 µM DEX or vehicle for 24 h. Thereafter, calvarial bones were used for RNA isolation as described below.

### Alkaline phosphatase (ALP) assay

Cells were lysed in 100 µl lysis buffer (1.5 mM Tris-HCl pH 7, 1 mM ZnCl_2_, 1 mM MgCl_2_, 1% triton X-100) and centrifuged for 15 min at 6,000 rpm at 4 °C. Aliquots of each sample were incubated with 100 µl ALP substrate buffer (100 mM diethanolamine, 0.1% triton X-100 supplemented with 1:10 37 mM p-nitrophenylphosphate) for 30 min at 37 °C. The enzymatic reaction was stopped with 40 mM NaOH, measured at a wavelength of 405 nm, and normalized to the total protein content determined by the BCA method from the same protein extracts.

### Promoter reporter assay

The GR binding site was identified as a transcriptional regulatory motif of the *Wnt16* promoter region by the online search tool (http://RegRNA.mbc.NCTU.edu.tw/; http://tfbind.hgc.jp/) and the eukaryotic promoter database (http://epd.vital-it.ch/). In order to investigate activation of the *Wnt16* promoter by GR signalling, a dual-reporter assay using secreted *Gaussia princeps* luciferase (GLuc) under the control of a *Wnt16* promoter was applied (pEZX-PG04, Gene Copeia). The plasmid pEZX-PG04 also contains secreted alkaline phosphatase (SEAP) as a secondary reporter gene for transfection normalization.

MC3T3-E1 cells were seeded in a density of 1.5 × 10^4^ cells per cm^2^ in basal medium (α-MEM, 10% FCS, 2 mM glutamine and 1% PenStrep). When cells were 70–80% confluent, medium was changed to basal medium without antibiotics one hour before the transfection. pEZX-PG04 was diluted to a final concentration of 1 μg plasmid DNA/100 μl medium (0.01 μg/μl) with the reduced serum medium Opti-Minimal Essential Medium (Life Technologies). Transient transfections were performed using the liposome-mediated *X-tremeGENE HP DNA* (Roche) transfection reagent. The medium was changed to fresh standard basal medium 24 h after transfection and DEX (1 µM) treatment was performed. Supernatant was harvested for the GLuc and SEAP assay 24 and 48 h after the treatment and stored at −20 °C until usage. Luciferase activity was measured by using the *Secrete-Pair Luminescence assay kit* (Gene Copeia) according to the manufacturer´s high sensitivity protocol.

### RNA isolation, RT and real-time PCR

RNA was extracted from mice hind legs by crushing them in liquid nitrogen and collecting the bone powder in Trifast (Peqlab, Erlangen, Germany). RNA from cultured cells was extracted by removing the medium and adding 1 mL Trifast to one well of a 6-well plate. RNA isolation was performed according to the manufacturer’s protocol. Five-hundred ng RNA were reverse transcribed using Superscript II (Life Technologies, Germany) and subsequently used for SYBR green-based real-time PCR reactions using a standard protocol (Applied Biosystems, USA). Primer sequences were used as follows: mu β-actin (forward, 5′-GATCTGGCACCACACCTTCT-3′; reverse, 5′-TCCTGGGAGTGTAGGTTTGG-3′); mu Wnt16 (forward, 5′-GGAGCTGTGCAAGAGGAAAC-3′; reverse,5′-AGTGGCGACCATACAGTTCC-3′); mu Ocn (forward, 5′- GCGCTCTGTCTCTCTGACCT-3′; reverse 5′- ACCTTATTGCCCTCCTGCTT-3′); mu ALP (forward, 5′-CTACTTGTGTGGCGTGAAGG-3′; reverse, 5′- CTGGTGGCATCTCGTTATCC-3′). PCR conditions were 50 °C for 2 min and 95 °C for 10 min followed by 40 cycles with 95 °C for 15 s and 60 °C for 1 min. The melting curve as assessed in the following program: 95 °C for 15 s, 60 °C for 1 min and 95 °C for 30 s. The results were calculated applying the ΔΔCT method and are presented in x-fold increase relative to β-actin.

### Western Blot analysis

BMSCs were isolated from hind legs of wild type mice and seeded at a density of 1 Mio. cells per cm² in DMEM containing 10% fetal FCS and 1% PenStrep. At 70% confluence, cells were switched to starving medium overnight. Cells were then treated with DEX (100 nM) for 48 hours. After removal of cell culture media, cells were lysed in 1 × RIPA lysis buffer (Cell Signaling Technology, Danvers, USA) and frozen at −80 °C. After thawing, samples were vortexed and centrifuged at 25,000 × g for 30 min at 4 °C. For Western Blot analysis, lysates were boiled for 5 min at 98 °C. Twenty μg of proteins were loaded on a 10% SDS-PAGE and transferred onto a 0.2 μm nitrocellulose membrane. After blocking for 1 h with 5% milk powder in Tris-buffered saline with 1% Tween-20 (TBS-T), membranes were incubated with antibodies against Wnt16 (1:500, Abcam, #ab109437) and GAPDH (1:2,000, Hytest, #5G4) overnight. After washing 3 times with TBS-T, incubation with an appropriate HRP-conjugated IgG antibody (1:2,000) for 1 h at RT followed. Proteins were visualized using the ECL Western-Blotting Substrate (Thermo Scientific). The chemiluminescence signal was recorded using a MF-*ChemiBIS 3*.*2bio imager system* (Biostep).

### Statistical analysis

Results are presented as means ± standard deviation (SD). Statistical evaluations were performed using a Student’s *t*-test, one-way or two-way ANOVAs (with Dunn’s multiple comparison corrections) as indicated in the figure legends. P-values ≤ 0.05 were considered statistically significant.

### Data availability

No large datasets were generated or analysed during this study. All other datasets analysed during this study are available from the corresponding author on reasonable request.

## Results

### *Wnt16* is downregulated by glucocorticoids in osteoblasts

**Figure 1:**
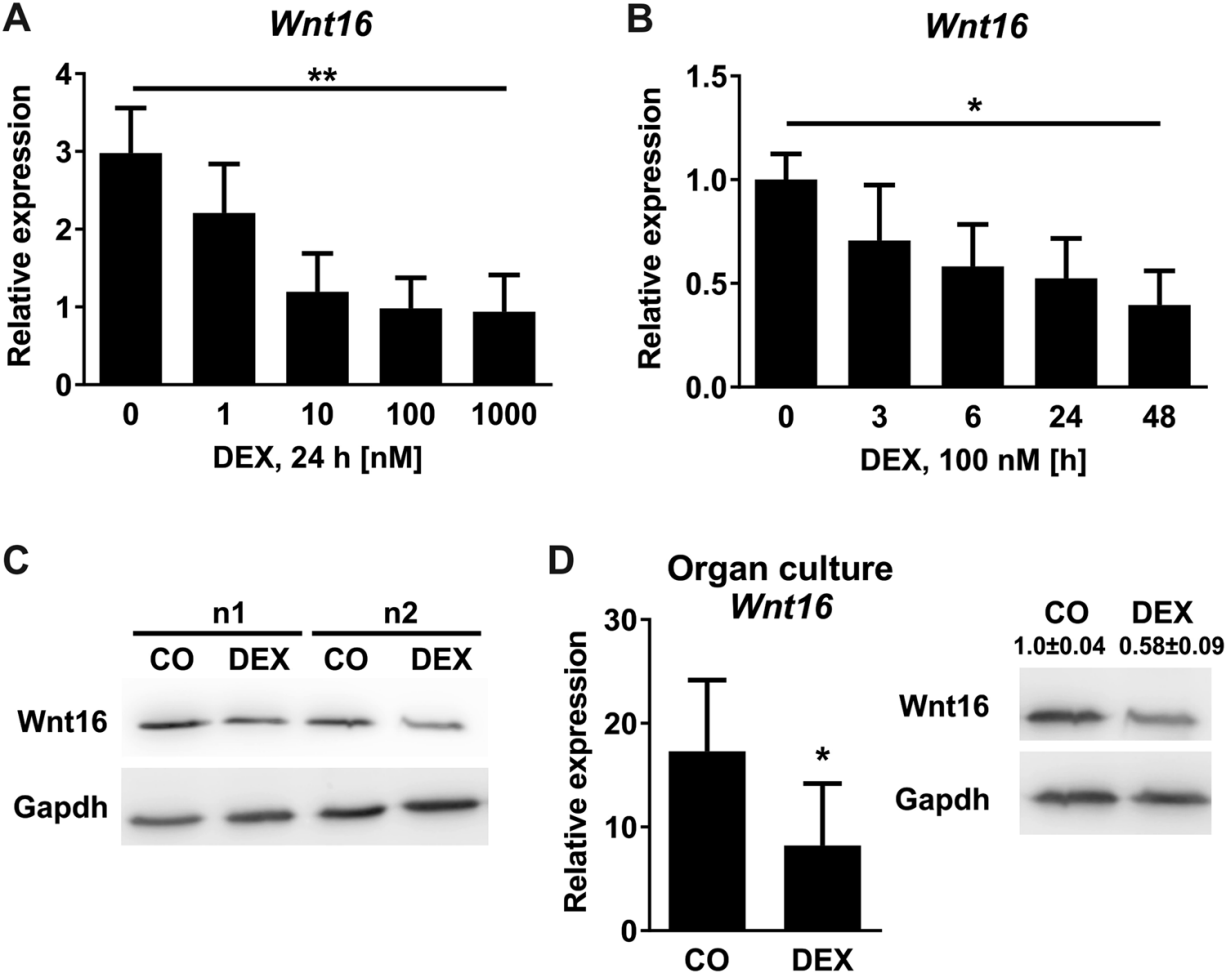
Glucocorticoids suppress Wnt16 *in vitro*. (**A**–**C**) Bone marrow stromal cells derived from wildtype mice were differentiated towards osteoblasts for 7 days and treated with (**A**) various concentrations of dexamethasone (DEX) and (**B**) various durations. N = 4–12. Wnt16 gene expression was quantified using real-time PCR. Beta-actin was used as housekeeping gene. (**C**) Cells were treated with 100 nM DEX or 48 h. Protein levels of Wnt16 and Gapdh were detected using Western blot. Two independent experiments are shown out of four. Each Western blot represents an own gel. Full gels are shown in Suppl. Fig. 1. (**D**) Calvarial bone from 3–5 days old C57BL/6 pups were cultured for 24 h in the presence or absence of 1 µM DEX. Wnt16 gene expression was quantified using real-time PCR (n = 6); protein expression using Western blot (n = 4). Numbers indicate semi-quantification of Western blots. Each Western blot represents an own gel. Full gels are shown in Suppl. Fig. 1. *p < 0.05; **p < 0.01 using One-way ANOVA for (**A**,**B**) and *t*-test for (**D**).

To investigate the effects of GCs on Wnt16 in osteoblasts, we treated murine osteoblasts derived from bone marrow stromal cells (mBMSCs) with increasing concentrations of DEX for different time periods. DEX led to a dose-dependent reduction of *Wnt16* expression, reaching a maximum at concentrations of 0.1 µM and 1 µM DEX, respectively (3.2-fold) (Fig. 1A). Furthermore, DEX suppressed *Wnt16* expression in osteoblasts in a time-dependent manner with the strongest reduction after 48 h (2.5-fold) (Fig. 1B). The suppression of Wnt16 after DEX treatment was also apparent at protein level (Fig. 1C).

In order to further validate inhibition of *Wnt16* expression by GCs we cultured calvaria of 3–5 day old mice with DEX for 24 h. Gene and protein expression analysis confirmed the inhibition of Wnt16 by GCs (Fig. 1D). DEX decreased *Wnt16* mRNA levels and Wnt16 protein expression more than 2-fold than in the control.

### Glucocorticoids reduce bone formation and *Wnt16* expression in a model of GIO

**Figure 2:**
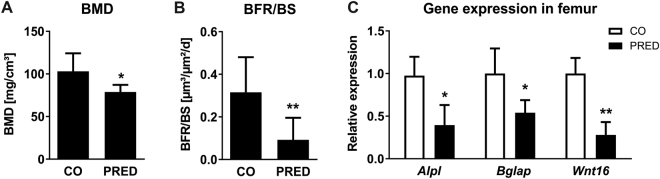
Glucocorticoids reduce bone formation and Wnt16 expression *in vivo*. (**A**–**C**) 24-week-old female FVB/N mice received slow-release pellets with prednisolone (PRED) or placebo (CO) for 4 weeks (3.5 mg; 60 day-release). (**A**) Bone mineral density (BMD) of the femoral bone was determined by using µCT and (**B**) bone formation rate per bone surface (BFR/BS) was quantified by dual calcein labeling following histomorphometry. (**C**) Gene expression levels of osteoblastic markers and *Wnt16* was measured in the femoral bone of prednisolone treated mice by real-time PCR and normalized to untreated mice. N = 6. *p < 0.05; **p < 0.01 using *t*-tests.

To assess the effects of GCs on *Wnt16* expression *in vivo*, we treated wildtype mice with the clinically used GC prednisolone for 4 weeks. As expected, bone mineral density of femoral bone was significantly reduced by 25% (Fig. 2A). In line, prednisolone exposure inhibited bone formation by more than 70% as determined by dynamic histomorphometry following dual calcein labelling (Fig. 2B). Accordingly, gene expression levels of osteoblastic markers in the femur of prednisolone-treated mice revealed a more than 2-fold downregulation of alkaline phosphatase (*Alpl*) and osteocalcin (*Bglap*) (Fig. 2C). In addition, expression of *Wnt16* mRNA was diminished by more than 70% in the femur of prednisolone-treated mice (Fig. 2C). A similar suppression of bone formation and *Wnt16* expression was observed in mice that were treated for 2 weeks with a higher dose of prednisolone (data not shown). Thus, these data indicate that *Wnt16* expression is inhibited by GCs *in vitro* and *in vivo*.

### *Wnt16* regulation by glucocorticoids is mediated by the GR and requires intact dimerization of the GR

**Figure 3:**
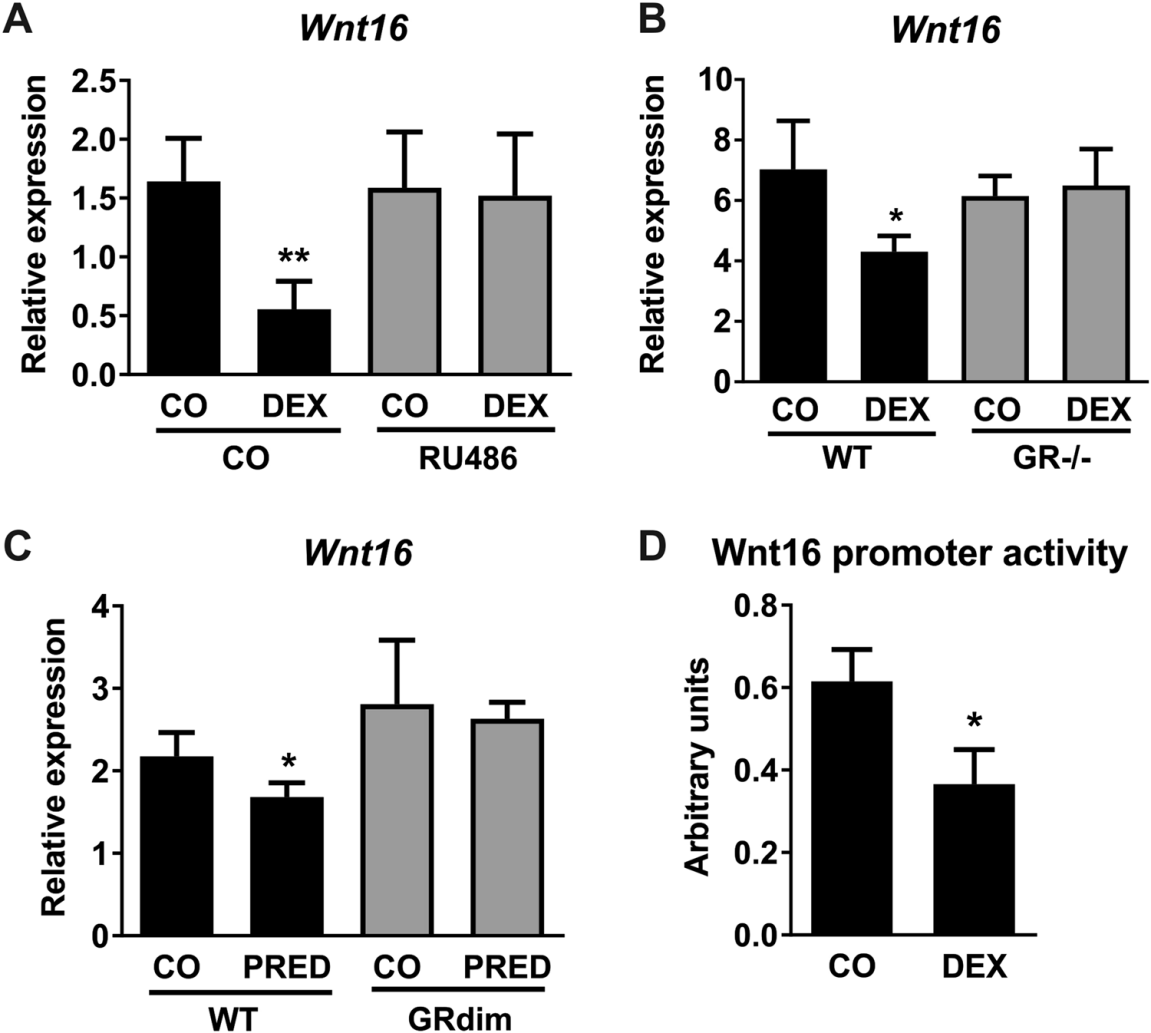
Regulation of Wnt16 by glucocorticoids is mediated by DNA-binding mechanisms of the GR. (**A**) Bone marrow stromal cells derived from wildtype mice were differentiated towards osteoblasts for 7 days and treated with 1 µM DEX with or without addition of 1 µM RU-486, a GR antagonist. *Wnt16* gene expression was measured by quantitative real-time PCR. N = 4–5. (**B**) GR-deficient osteoblasts were treated with 1 µM DEX and Wnt16 expression was determined by real-time PCR. N = 3. (**C**) 10-week-old female FVB/N wildtype and GR dimerization-deficient mice received slow-release pellets with prednisolone (PRED, 15 mg; 60 day-release) for 14 days and *Wnt16* expression of the femoral bone was assessed by real-time PCR. N = 3. (**D**) MC3T3-E1 cells were transfected with the *Wnt16* promoter reporter construct (pEZX-PG04) containing Gaussia princeps luciferase (GLuc) under the control of a *Wnt16* promoter and treated with 1 µM DEX. After 48 h the activity of GLuc was measured in the supernatants by luminescence. Values were normalized to secreted alkaline phosphatase and control (CO). N = 3. *p < 0.05; **p < 0.01 using two-way ANOVAs.

We next elucidated the underlying mechanisms of GC-induced Wnt16 regulation. Since GCs mediate their effects via the GR, we first co-treated osteoblasts with DEX and the GR-antagonist RU-486. Application of RU-486 completely abolished the DEX-induced reduction of *Wnt16* expression (Fig. 3A). RU-486 alone had no effect on *Wnt16* mRNA levels. To confirm the involvement of the GR in mediating GC-induced effects on *Wnt16* expression, we treated osteoblasts derived from GR-deficient mice (*GR*^*−/−*^ mice) with DEX. Whereas DEX reduced *Wnt16* expression in osteoblasts derived from wildtype mice, DEX failed to suppress *Wnt16* in the absence of the GR (Fig. 3B).

The GR alters gene expression via different modes of action, including binding as a homodimer to GREs in the promotor of GC-target genes and interaction as a monomer with DNA-bound transcription factors such as NF-κB, AP-1, IRF-3 or STAT5^46^. To further study the underlying mode of GR action in the regulation of Wnt16, we analysed mice with attenuated GR dimerization (*GR*^*dim*^ mice^47^) that show reduced DNA binding as revealed by genome wide studies^48^. GR^dim^ mice were treated with prednisolone for 14 days. In contrast to wildtype littermate control mice, in which prednisolone treatment reduced *Wnt16* expression in femoral bone, prednisolone did not reduce skeletal *Wnt16* expression in *GR*^*dim*^ mice (Fig. 3C). Thus, intact GR dimerization and likely DNA binding of the GR is required for mediating GC effects on *Wnt16* expression.

As these results suggested an involvement of direct DNA-binding mechanisms of the GR, we next searched for transcriptional GR regulatory motifs in the promotor of *Wnt16* with the help of online search tools. Using three different websites, we identified multiple putative GR binding sites. Further, we performed a luciferase dual-reporter assay to verify the regulation of Wnt16 promoter activity via GCs. Following lipid-mediated transfection of the Wnt16 promotor construct in the osteoblastic cell line MC3T3-E1, we treated the cells for 48 h with DEX. Luciferase activity was reduced up to 30% in DEX-treated cells (Fig. 3D) indicating that the GR directly binds GRE motifs in the Wnt16 promoter region.

### Wnt16 rescues GC-induced suppression of bone formation

**Figure 4:**
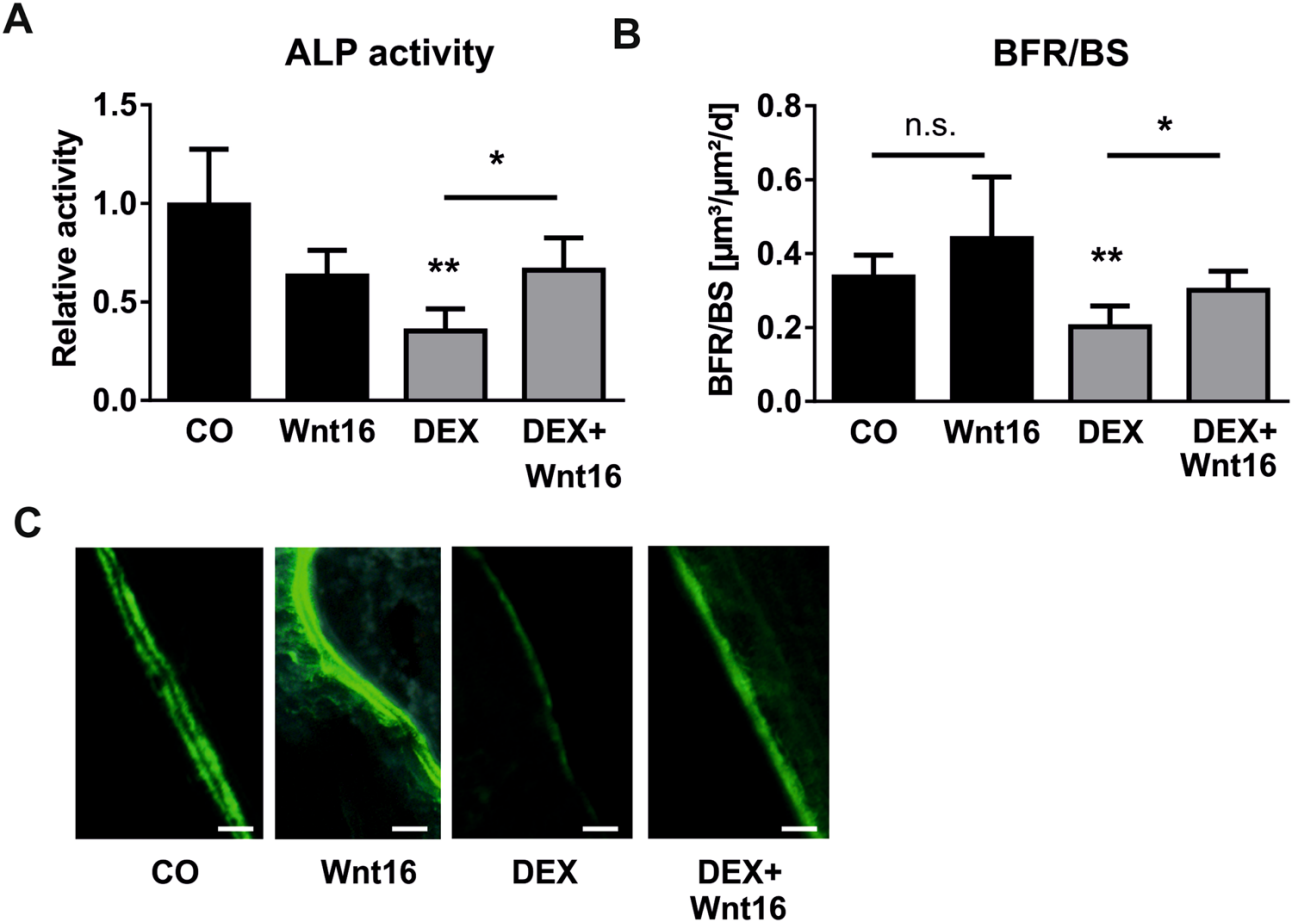
Wnt16 counteracts GC-induced suppression of bone formation. (**A**) Bone marrow stromal cells derived from wildtype mice were differentiated towards osteoblasts for 7 days and treated with 1 µM DEX, 1 µg/ml Wnt16 or both, respectively. Osteoblast function was assessed by measuring alkaline phosphatase (ALP) activity. N = 4–5. (B + C) 12-week-old C57BL/6 mice were treated with 0.5 mg/kg DEX alone or in combination with 500 ng recombinant Wnt16 for 3 weeks subcutaneously over the calvaria. (**B**) Bone formation rate per bone surface (BFR/BS) was measured by dual calcein labeling and subsequent histomorphometric analysis. (**C**) Representative calcein labels per group. Scale bar: 20 µm. N = 3–7. *p < 0.05; **p < 0.01 using two-way ANOVAs.

To assess the functional role of Wnt16 in GC-induced suppression of osteoblast function and bone formation, we treated osteoblasts with DEX and/or recombinant Wnt16 and measured alkaline phosphatase (ALP) activity. As shown in Fig. 4A, ALP activity was reduced more than 2-fold upon DEX treatment, but addition of Wnt16 abrogated GC-induced suppression of osteoblast function. To further investigate the involvement of Wnt16 in GC-mediated inhibition of bone formation *in vivo*, we treated wildtype mice with DEX and recombinant Wnt16, respectively, for 3 weeks by subcutaneous injection over the calvaria and determined the bone formation rate by dual calcein labelling. Histomorphometric analysis revealed a strong reduction in bone formation in DEX-treated animals. The concomitant treatment with Wnt16, however, rescued the DEX-induced repression of bone formation (Fig. 4B,C). Thus, Wnt16 is involved in GC-induced suppression of bone formation *in vivo*.

## Discussion

GCs are frequently used to treat chronic inflammation. However, long-term GC treatment causes severe side effects on the skeleton and leads to an increased fracture risk mainly due to the sustained suppression of bone formation^11^. Recently Wnt16 has been linked to cortical bone thickness and fracture risk^28–31,33^ and has been recognized as a positive regulator of bone mass and strength^32,34–36^. In this study, we investigated whether Wnt16 is a target of GC action. We show that GCs suppress Wnt16 expression *in vitro* and *in vivo* and that Wnt16 exposure counteracts the inhibitory effects of GCs on osteoblast function.

Using primary mouse osteoblasts, we found that DEX reduces *Wnt16* expression in a time- and dose-dependent manner. Moreover, DEX treatment also reduced Wnt16 protein levels in osteoblasts and calvaria organ cultures. These results are in line with other studies that have shown that increasing GC doses oppose Wnt signalling thereby attenuating osteoblast differentiation and function. In a study with murine osteoblasts, high doses of corticosterone reduced the expression of *Wnt7b* and *Wnt10*^42^. In contrast to *Wnt7b* and *Wnt10b*, *Wnt16* was not modulated by GCs in a biphasic fashion as also low doses negatively affected *Wnt16* expression.

In order to investigate the effects of GCs on Wnt16 *in vivo*, a GIO mouse model was used. Treatment of mice with prednisolone for four weeks caused a reduction of BMD and inhibition of bone formation as described previously^13,18^. In this model, *Wnt16* expression was reduced in the bone tissue of GC-treated mice, and coincided with the reduced expression of the pro-osteoblastic markers alkaline phosphatase and osteocalcin.

After showing that Wnt16 is a novel target of GC action in bone, we analysed the underlying molecular mechanism. GCs bind to the GR and mediate their function by binding as a homodimer to palindromic DNA sequences (so called GRE, glucocorticoid response elements), binding to GRE half-sites as a monomer or binding as a monomer to DNA-bound transcription factors and thereby modulate target gene transcription^49^. Whereas for most anti-inflammatory GC effects GR dimerization is essential^50–52^, suppression of bone formation is proposed to be mediated by GR monomers interacting with the transcription factor AP-1^13^. Our data show that the suppression of *Wnt16* expression by GCs is dependent on GR dimerization and DNA binding of the GR complex. This is in contrast to GC-induced suppression of other osteoblastic genes and cytokines such as *Runx2*, *Col1a1* or *Il11*, which is likely mediated by monomeric GR transrepression mechanisms^13^. However, a distinct mechanism of GC-induced direct transrepression via binding of GR to evolutionary-conserved cis-acting negative GC-responsive elements (IR nGREs) was described by Surjit *et al*.^53^. In contrast to tethered transrepression, nGRE-mediated gene repression is abolished by the *GR*^*dim*^ mutation. Moreover, co-treatment with RU486 prevented GC-induced IR GRE-mediated direct transrepression^53^. Thus, it is tempting to speculate that the *Wnt16* gene may also contain IR nGREs, especially as also other GC-regulated Wnt genes such as *Wnt1*, *Wnt5a* and *Wnt10b* contain putative IR nGREs^53^ and the number of genes regulated by nGREs has been proposed to be higher than previously thought. Additional evidence for the requirement of DNA-binding mechanisms of the GR is provided by *in silico* analyses, which identified GR transcriptional regulatory motifs in the *Wnt16* promoter region, and a promoter assay, which revealed a suppression of *Wnt16* promoter activity after GC treatment. Finally, considering that *Wnt16* expression was already regulated after 3 hours, our data suggest that *Wnt16* is a primary response gene of GC signalling via direct GR DNA-binding mechanisms in osteoblasts. Further studies are necessary to explore the exact GR binding motifs within the putative negative GREs on the *Wnt16* promoter.

After having established the regulatory role of GCs on *Wnt16* expression, we addressed whether Wnt16 is able to rescue GC-induced suppression of bone formation. Interestingly, single treatment of both, primary osteoblasts and mice did not significantly alter osteoblast function, indicating that short-term treatment with Wnt16 may not be sufficient to enhance osteoblast function in the same way as long-term exposure as in WNT16 overexpressing mice^36,37^. Similarly, osteoblasts derived from Wnt16KO mice did not show alterations in their differentiation capacity^34^ However, co-treatment of osteoblasts with DEX and recombinant Wnt16 abrogated the GC-induced suppression of osteoblast function *in vitro* and *in vivo*. This novel finding suggests that restoring *Wnt16* expression can at least partly abolish GC-induced suppression of bone formation *in vitro* and *in vivo*. One limitation of this study is the local treatment with DEX and Wnt16, despite GIO being a systemic disease. However, considering that Wnt ligands act in a local, paracrine fashion, we chose the local treatment of calvarial bone with DEX and Wnt16. This approach has also been taken in a previous study, in which inflammation-induced calvarial bone loss was rescued using recombinant Wnt16^34^. Future studies using Wnt16 overexpressing mice are underway that may provide more insights into the systemic rescue of GIO via Wnt16^54,55^.

Collectively, our data suggest that Wnt16 is a direct target of GC action and that restoration of Wnt16 alleviates GC-suppressed bone formation. Thus, Wnt16 may represent a novel target for therapeutic intervention in GIO.

## Electronic supplementary material

Supplementary information
