# Author Correction: Glucocorticoids suppress Wnt16 expression in osteoblasts *in vitro* and *in vivo*

**DOI:** 10.1038/s41598-020-65962-6

**Published:** 2020-06-04

**Authors:** Susanne Hildebrandt, Ulrike Baschant, Sylvia Thiele, Jan Tuckermann, Lorenz C. Hofbauer, Martina Rauner

**Affiliations:** 1 Division of Endocrinology, Diabetes, and Bone Diseases, Department of Medicine III, Technische Universität Dresden, Dresden, Germany; 2 Center for Healthy Aging, Technische Universität Dresden, Dresden, Germany; 3 Institute of Comparative Molecular Endocrinology (CME), University of Ulm, Ulm, Germany; 4 Present Address: Institute of Chemistry and Biochemistry, Freie Universität Berlin, Berlin, Germany

Correction to: *Scientific Reports* 10.1038/s41598-018-26300-z, published online 07 June 2018

This Article contains an error. The text in Figure 2’s legend,

“Bone mineral density (BMD) of the femoral bone was determined by using µCT…”

should read:

“Bone mineral density (BMD) of the femoral bone was determined by using pQCT…”

