# Supplementary material for: Glucocorticoids suppress Wnt16 expression in osteoblasts in vitro and in vivo
Source: Sci Rep. 2018 Jun 7;8:8711. doi: 10.1038/s41598-018-26300-z (PMC5992207; doi:10.1038/s41598-018-26300-z)

SUPPLEMENTARY INFORMATION

Glucocorticoids suppress Wnt16 expression in osteoblasts *in vitro* and *in vivo*

Susanne Hildebrandt[1-2 *#], Ulrike Baschant[1-2 *], Sylvia Thiele[1-2], Jan Tuckermann[3],

Lorenz C. Hofbauer[1-2], Martina Rauner[1-2]

[1]Division of Endocrinology, Diabetes, and Bone Diseases; Department of Medicine III & [2]Center for Healthy Aging, Technische Universität Dresden, Germany; [3]Institute of Comparative Molecular Endocrinology (CME), University of Ulm, Germany.

# Current address: Institute of Chemistry and Biochemistry, Freie Universität Berlin, Berlin, Germany

* Authors contributed equally.

Supplementary Figure 1. Full Western blot images from Figure 1. Bone marrow stromal cells derived from wildtype mice were differentiated towards osteoblasts for 7 days and treated with dexamethasone (DEX) or vehicle (CO) for 48 hours. Wnt16 and GAPDH were analyzed. Numbers on the left indicate molecular weight (kDa).

Fig. 1C

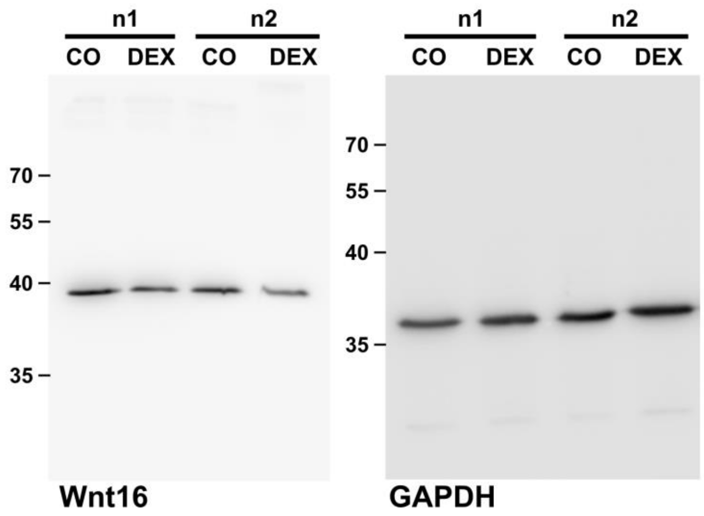

Fig. 1D

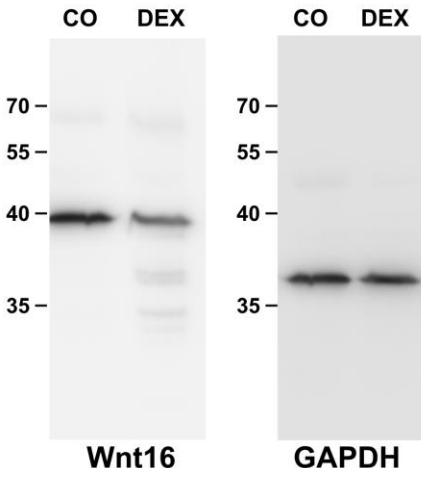

Supplement: Supplementary file 1 — Supplementary information [file 41598_2018_26300_MOESM1_ESM.pdf]
